# Metabolomic Analysis Reveals Extended Metabolic Consequences of Marginal Vitamin B-6 Deficiency in Healthy Human Subjects

**DOI:** 10.1371/journal.pone.0063544

**Published:** 2013-06-11

**Authors:** Jesse F. Gregory, Youngja Park, Yvonne Lamers, Nirmalya Bandyopadhyay, Yueh-Yun Chi, Kichen Lee, Steven Kim, Vanessa da Silva, Nikolas Hove, Sanjay Ranka, Tamer Kahveci, Keith E. Muller, Robert D. Stevens, Christopher B. Newgard, Peter W. Stacpoole, Dean P. Jones

**Affiliations:** 1 Food Science and Human Nutrition Department, Institute of Food and Agricultural Sciences, University of Florida, Gainesville, Florida, United States of America; 2 Clinical Biomarkers Laboratory, Division of Pulmonary, Allergy and Critical Care Medicine, Department of Medicine and Center for Clinical and Molecular Nutrition, Department of Medicine, Emory University, Atlanta, Georgia, United States of America; 3 Computer and Information Science and Engineering, University of Florida, Gainesville, Florida, United States of America; 4 Department of Biostatistics, University of Florida, Gainesville, Florida, United States of America; 5 Industrial Engineering Department, Hanyang University, Seoul, Korea; 6 Department of Health Outcomes and Policy, University of Florida, Gainesville, Florida, United States of America; 7 Sarah W. Stedman Nutrition and Metabolism Center, Duke University Medical Center, Durham, North Carolina, United States of America; 8 Division of Endocrinology and Metabolism, Department of Medicine, University of Florida, Gainesville, Florida, United States of America; 9 Department of Biochemistry and Molecular Biology, College of Medicine, University of Florida, Gainesville, Florida, United States of America; Paris Institute of Technology for Life, Food and Environmental Sciences, France

## Abstract

Marginal deficiency of vitamin B-6 is common among segments of the population worldwide. Because pyridoxal 5′-phosphate (PLP) serves as a coenzyme in the metabolism of amino acids, carbohydrates, organic acids, and neurotransmitters, as well as in aspects of one-carbon metabolism, vitamin B-6 deficiency could have many effects. Healthy men and women (age: 20-40 y; n = 23) were fed a 2-day controlled, nutritionally adequate diet followed by a 28-day low-vitamin B-6 diet (<0.5 mg/d) to induce marginal deficiency, as reflected by a decline of plasma PLP from 52.6±14.1 (mean ± SD) to 21.5±4.6 nmol/L (P<0.0001) and increased cystathionine from 131±65 to 199±56 nmol/L (P<0.001). Fasting plasma samples obtained before and after vitamin B6 restriction were analyzed by ^1^H-NMR with and without filtration and by targeted quantitative analysis by mass spectrometry (MS). Multilevel partial least squares-discriminant analysis and S-plots of NMR spectra showed that NMR is effective in classifying samples according to vitamin B-6 status and identified discriminating features. NMR spectral features of selected metabolites indicated that vitamin B-6 restriction significantly increased the ratios of glutamine/glutamate and 2-oxoglutarate/glutamate (P<0.001) and tended to increase concentrations of acetate, pyruvate, and trimethylamine-N-oxide (adjusted P<0.05). Tandem MS showed significantly greater plasma proline after vitamin B-6 restriction (adjusted P<0.05), but there were no effects on the profile of 14 other amino acids and 45 acylcarnitines. These findings demonstrate that marginal vitamin B-6 deficiency has widespread metabolic perturbations and illustrate the utility of metabolomics in evaluating complex effects of altered vitamin B-6 intake.

## Introduction

Vitamin B-6 exists in many dietary sources, yet an individual's particular food consumption pattern and certain drug-nutrient interactions can lead to low vitamin B6 status. The vitamin B-6 status of much of the United States population is adequate. However, the lower percentiles of intake are associated with low vitamin B-6 status [1], [2] that is more prevalent in smokers, women and the elderly [1], [3], [4]. Many inflammatory conditions also are associated with lower vitamin B-6 status regardless of intake [5], [6], but the mechanism is unknown. The use of certain common drugs such as theophylline [7] and oral contraceptive agents [1], [8] also is associated with reduced vitamin B-6 status.

The coenzymic form of vitamin B-6, pyridoxal phosphate (PLP), serves as a coenzyme for over 140 enzymes in human metabolism. PLP is thus involved in a wide array of functions [9] including: the catabolism and interconversion of most amino acids; the formation of various organic acids, including species involved in the TCA cycle and gluconeogenesis; heme synthesis; and several key steps in pathways associated with one-carbon metabolism. Vitamin B-6 deficiency also is associated with interconversions of long-chain polyunsaturated fatty acids.

Plasma PLP concentration of <20 nmol/L reflects vitamin B-6 deficiency [3], while 20–30 nmol/L indicates marginal status [10], [11]. The consequences of marginal deficiency are unclear, but chronically low vitamin B6 status is associated with increased risk of cardiovascular disease [12]–[17], deep-vein thrombosis [18]–[20], stroke [21] and certain cancers [22], [23]. The mechanisms responsible for these disease connections are unknown but do not appear to be associated with hyperhomocysteinemia [5]. In view of the many coenzymic roles of PLP, further investigation of the in vivo metabolic consequences of inadequate vitamin B6 status may provide better insight into the effects of marginal vitamin B-6 deficiency.

We have investigated the consequences of inadequate vitamin B6 status using a series of protocols that involve the use of controlled low-vitamin B-6 diets in healthy volunteers [24]–[29]. In these studies, we employed targeted metabolite profiling and in vivo stable isotope tracer kinetic protocols to derive functional information about specific vitamin-dependent processes in one-carbon metabolism and related pathways while the participants were in adequate and marginal vitamin B6 status. These studies led to the following major observations concerning the effects of vitamin B-6 restriction: (a) surprising resiliency of one-carbon metabolism to effects of vitamin B6 deficiency, (b) changes glycine kinetics and concentration, (c) the resiliency of transsulfuration flux concurrent with an expansion of the cystathionine pool, (d) individual variability in the kinetics of glutathione synthesis, and (e) altered patterns of circulating n-3 and n-6 polyunsaturated fatty acids [24]–[29]. This work has led to new insights into PLP-dependent metabolic processes and the influence of vitamin B6 nutritional status.

Advances in both NMR and mass spectral aspects of metabolomics have impacted many facets of biology including the nutritional sciences [30], [31]. The nutritional applications of NMR metabolomics to date have tended to focus on dietary effects on macronutrient metabolism and intermediary metabolites (for example, [32], [33], with few applications of these powerful tools in characterizing the metabolic effects varying levels of micronutrient status (for example, [34]). The direct analysis of plasma or urine by NMR provides a useful approach that complements mass spectrometry for evaluating metabolic phenotypes associated with nutritional adequacy and deficiency and for evaluating nutrient-gene and nutrient-disease interactions.

The study reported here was conducted to investigate the impact of controlled vitamin B-6 depletion through the use of ^1^H-NMR analysis of plasma from 23 healthy participants from two recent vitamin B-6 restriction studies [27], [28]. We examined NMR spectra of intact plasma with and without deproteination by filtration as an untargeted means of evaluating vitamin B6-dependent changes in plasma constituents. The results were evaluated using multivariate analysis accounting for the paired structure of the data to assess differences in spectral patterns and metabolite profiles. On a subset of these samples (n = 18) we also performed provisional quantification on selected metabolites by spectral curve fitting (Chenomx). Because of the critical role of vitamin B6 in the metabolism of amino acids and organic acids, we also conducted targeted metabolite profile analysis of amino acids, selected organic acids and acylcarnitines using quantitative analyses of NMR signals, and mass spectrometry.

## Materials and Methods

### Participants and nutritional protocols

The blood samples for this study were obtained as described previously [27], [28] from healthy men (n = 12) and women (n = 11), aged 20–40 y, who participated in two identical dietary vitamin B6 restriction protocols designed to assess the metabolic effects of marginal vitamin B6 status. Health was determined by physical examination and routine tests of hepatic, renal, thyroid and hematological function [27], 28. No participant had a history of gastrointestinal surgery, chronic disease, smoking or chronic drug use or alcoholism, body mass index not greater than 28 kg/m^2^, vitamin, amino acid, or protein supplementation, or chronic consumption of a high-protein diet. Nutritional adequacy was indicated normal concentrations of serum folate (greater than 7 nmol/L), serum vitamin B-12 (greater than 200 pmol/L), plasma PLP (greater than 30 nmol/L), and plasma total homocysteine (less than 12 µmol/L). All participants gave written informed consent. The study was approved by the University of Florida Institutional Review Board and the UF Clinical Research Center (CRC) Scientific Advisory Committee. This study was registered at clinicaltrials.gov as NCT00877812.

The experimental protocol consisted of two dietary periods with a metabolic assessment of tracer kinetics and blood sampling for metabolite profiling at the end of each [27], [28]. The first consisted of a 2-d nutritionally adequate controlled diet (total vitamin B-6 = 1.02±0.11 mg/d), followed by the first metabolic assessment of participants in documented adequate vitamin B6 status. The second period involved a 28-d low vitamin B-6 diet to induce a state of marginal vitamin B-6 deficiency (PLP ∼20 nmol/L), followed immediately by a metabolic assessment identical to the first. The vitamin B-6 restriction diet (total vitamin B-6 = 0.37±0.04 mg/d) provided total protein intake of 1 g kg^−1^ d^−1^ and mean methionine and cystine intake of 21 and 17 mg kg^−1^ d^−1^, respectively. During both metabolic assessments, blood samples analyzed in this metabolomic analysis were obtained in the morning after withholding food overnight and were followed by primed, constant infusion protocols reported previously [27], [28]. The analyses reported here were performed on plasma obtained before and after the 28-d vitamin B-6 restriction period. The samples taken for NMR and mass spectral analysis were drawn by syringe from an indwelling catheter in an antecubital vein, mixed gently in a sodium EDTA tube, cooled on ice, centrifuged within 15 min of collection and then stored at −80°C.

### Targeted quantitative analysis

Measurement of plasma PLP [25], [35] and aminothiols (total glutathione, cysteine, and homocysteine; [36]) was performed by fluorometric HPLC. Plasma cystathionine, a sensitive functional biomarker of vitamin B-6 deficiency [26], [27], [37]), was determined by gas chromatography-mass spectrometry [38]. Plasma concentrations of free amino acids and acylcarnitines were determined by electrospray tandem mass spectrometry using isotope dilution methods [39]. We determined plasma glucose concentration using a commercial hexokinase-based assay kit (Sigma GAHK-20).

### NMR analysis

#### ^1^H-NMR spectroscopy

^1^H-NMR spectra were obtained at 600 MHz on a Varian INOVA 600 spectrometer with water presaturation at 25°C at the Emory University Clinical Biomarkers Laboratory, as described previously [32]. Plasma samples were analyzed in intact form (i.e., unfiltered) and following filtration through a 3 kD nominal cut-off membrane that removes proteins, lipids and lipoproteins as well as protein-bound forms of certain small molecules.

#### Unfiltered Plasma – ^1^H-NMR spectroscopy

Plasma samples were thawed and 600 μL was mixed with 66 μL of deuterium oxide (D_2_O) with TMS [3-(trimethylsilyl)-1-propanesulfonic acid sodium salt (C_6_H_15_NaO_3_SSi, 1% w/w)] as internal standard, pH 7.4–7.6. ^1^H-NMR spectra were measured at 600 MHz on a Varian INOVA 600 spectrometer with water presaturation at 25°C essentially as described [32], [40]. Preprocessing of spectra included baseline correction, spectral alignment, elimination of uninformative spectral regions and normalization [32], [40], with baseline correction and peak alignment [41], [42]. The water signal region (4.5–5 ppm) was eliminated because of variable suppression of the water signal, as were the regions between 5.4–6.7 ppm and 0–0.1 ppm that contained no significant metabolite signal [40], [43]. The spectral regions containing signals from EDTA and EDTA complexes with calcium and magnesium [44] also were excluded.

#### Filtered Plasma – ^1^H-NMR spectroscopy

We examined the spectra of plasma samples following ultrafiltration, which removed most proteins, lipoproteins and lipids, in order to facilitate evaluation of small molecules. Centrifugal ultrafiltration devices (Microcon YM-3, 3,000 nominal molecular weight cutoff, Millipore Corp.) were pre-washed by centrifuging three times for 15 min each with 500 μl of portions of distilled water to remove the glycerol preservative. Each plasma sample was transferred to a pre-washed device and centrifuged at 13,800×g for 60 min. Filtered plasma samples were mixed with TMS and adjusted pH 7.0–7.4. 1H-NMR spectra were acquired at 600 MHz on a Varian INOVA 600 spectrometer with water presaturation at 25°C as described above. As described for previous 1H-NMR studies of human plasma conducted by the Emory Clinical Biomarkers Laboratory [32], the identity of major signals was supported by the addition of standards (amino acids, organic acids, energy intermediates), comparisons to spectra in chemical databases, reference to a rigorous description of NMR spectra of human plasma [43], application of the Chenomx database, and confirmation with 2-dimensional (2-D) NMR techniques.

#### Quantitative ^1^H-NMR analysis of metabolites

The analysis of NMR spectra from filtered samples utilizing spectral fitting techniques (Chenomx, Edmonton, Alberta, Canada) allowed estimation of the concentrations of major plasma constituent small molecules [45], [46]. For 5 of the 23 participants, at least one of the NMR spectra did not meet the criteria needed for the Chenomx analysis. Therefore, quantitative analysis is reported only on the 18 participants for which pre- and post-vitamin B-6 restriction data were obtained. We report from this analysis estimates of the concentrations of 15 plasma constituents for these 18 participants before and after vitamin B-6 restriction.

### Data analysis

Principal Component Analysis (PCA) and Orthogonal Signal Correction-Partial Least Squares Discriminant Analysis (OPLS-DA) of processed spectra were performed using Pirouette software (Infometrix, Bothell, WA). PCA and OPLS-DA score plots and loading plots of NMR spectral data were used to visualize relational patterns and discriminatory factors to classify the groups. The OPLS-DA procedure, being a supervised technique (i.e., treatment groupings included in the analysis), removes variation not correlated to classification after centering the mean value for each frequency [33], [40], [47]. Data were evaluated for unfiltered and filtered plasma using score plots in OPLS-DA analysis to evaluate the classification according to vitamin B-6 status. For each discriminating spectral region, we assigned the compounds that possibly contributed on the basis of previous literature [43], experiments involving the addition of selected standards, comparison to spectra in databases or applications with 2-dimensional NMR procedures [32], [40].

To account for the paired data structure within our study (i.e., observations before and after vitamin B-6 restriction), we then conducted multilevel PLS-DA as described by Westerhuis et al. [38] as follows. First, the paired structure was created by averaging each ppm for all individual spectra including B6 baseline and restricted. From each individual, the average spectrum were created with B6 baseline and restricted. Secondly, for each subject, the table of correction factors for each ppm was extracted by dividing averaged intensity at each ppm from all individual spectra with averaged intensity at each ppm from each individual averaged spectrum. Thirdly, using correction factors for each ppm, the intensities for each B6 baseline and restricted were adjusted to generate paired data structure. Lastly OPLS-DA was run on the paired data structure. Additonal analysis to identify and visualize discriminatory spectral regions was conducted using S-plots of covariance versus correlation for spectral data [48] followed by loading plots of covariance versus chemical shift on mean spectra. After calculation of the top 5% of discriminatory loadings in this analysis (647 and 381 for unfiltered and filtered plasma, respectively), we visually selected points (65 and 26 for unfiltered and filtered, respectively) in regions of the S-plots that were clearly separated from the central risk zone. These represented the most highly discriminating features.

Further statistical analysis of metabolite concentrations was performed after log base 2 transformation using a recently developed method for global hypothesis testing in repeated measures high-dimensional data [49]. The log base 2 transformation was a means to meet the Gaussian requirement. For subsequent local hypothesis testing, we evaluated differences in the transformed concentration of individual plasma constituents using paired t-test and the positive false discovery rate method [50] to adjust p-values in anticipation of the inflation of false positive rate as a result of multiple testing. The effects of change in plasma PLP and change in plasma cystathionine were evaluated using the test for Pearson correlation coefficient in conjunction with the positive false discovery rate method to account for multiple testing.

Ratios of selected metabolites derived from NMR analysis were evaluated to assess product/precursor relationships before and after vitamin B-6 restriction. Asparagine/aspartic acid and pyruvate/alanine ratios served as static indicators of the proportions of amide versus acid forms of glutamate and aspartate. The ratios of pyruvate/alanine and 2-oxoglutarate/glutamate provided a measure of the product/precursor ratio for these aminotransferases.

A level of statistical significance of 0.05 was employed in all procedures.

## Results

### Efficacy of the vitamin B-6 restriction protocol

The dietary protocol provided a controlled, nutritionally adequate 2-day stabilization period followed by very low vitamin B-6 intake for 28 days, and this protocol is effective for selectively inducing a state of marginal vitamin B-6 deficiency in healthy adults [25], [51], [52]. Plasma PLP concentration declined from 52.6±14.1 (mean ± SD) to 21.5±4.6 nmol/L (P<0.0001), which indicated a change from adequate vitamin B-6 status to marginal deficiency. Additional descriptive data regarding the 23 participants in this protocol have been reported in part previously [27]–[29] and are summarized (Table S1).

### Metabolic effects of vitamin B-6 restriction

#### Global statistical analysis of ^1^H NMR spectral data

^1^H-NMR analysis of plasma samples yielded spectra that were qualitatively similar to those originally reported and annotated by Nicholson and colleagues [43], [53]. Filtration of plasma attenuated the regions of the spectrum attributable to lipids and proteins, as expected. Representative spectra of unfiltered and filtered plasma in vitamin B-6 adequate and restricted states are presented in Figure S1.

Statistical evaluation of spectra first involved assessing the change in overall spectral data (before versus after vitamin B-6 restriction) using a recent method for global hypothesis testing for high-dimensional repeated measures outcomes of pairwise data [49]. By this method, the overall change across the entire spectrum was not significant for unfiltered plasma (p-value = 0.432) and filtered plasma (p-value = 0.549). Gender also had no significant effect on the overall change in spectra of unfiltered plasma (p-value = 0.683) or filtered plasma (p-value = 0.275) in this analysis. Because this n = 23 data set was derived from two equivalent vitamin B-6 restriction trials, we also tested for effects of trial 1 versus trial 2. The effect of trial (1 versus 2) had no significant effect on the overall change for unfiltered plasma (p-value = 0.103) or filtered plasma (p-value = 0.346).

#### Multilevel PLS-DA evaluation of ^1^H-NMR spectra to assess effects of vitamin B-6 restriction in paired data

Principal Component Analysis that did not account for paired structure of the spectral data obtained before and after vitamin B-6 restriction showed no separation or grouping of data according to vitamin B-6 status for both unfiltered and filtered plasma (data not shown). OPLS-DA, which is a supervised technique in which treatment groupings are identified, yielded unambiguous classification of data patterns according to nutritional status (i.e., pre- and post-vitamin B-6 restriction) for both unfiltered and filtered plasma (not shown). This provided evidence of extended compositional differences associated with vitamin B-6 restriction, although the OPLS-DA method also did not account for the paired data structure.

**Figure 1:**
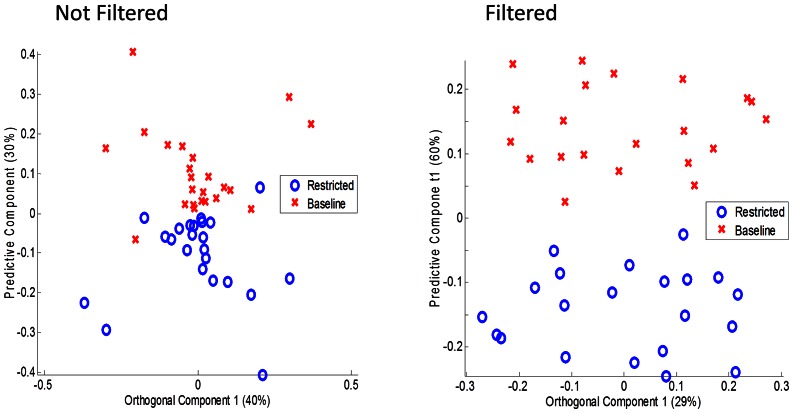
Metabolic patterns separated adequate vitamin B6 at baseline from marginally deficient status after 4 weeks on a restricted diet. Plasma samples from 23 healthy, young adults collected at baseline and after 4 weeks consuming a vitamin B6-restricted diet were examined by ^1^H-NMR spectroscopy followed by multilevel partial least square-discriminant analysis (multilevel PLS-DA). A) Score plot for unfiltered plasma, which contains relatively large signal from lipoproteins. B) Score plot for plasma filtered through a 3 μm pore size filter to remove most of the lipoprotein before ^1^H-NMR spectroscopy analysis.

**Figure 2:**
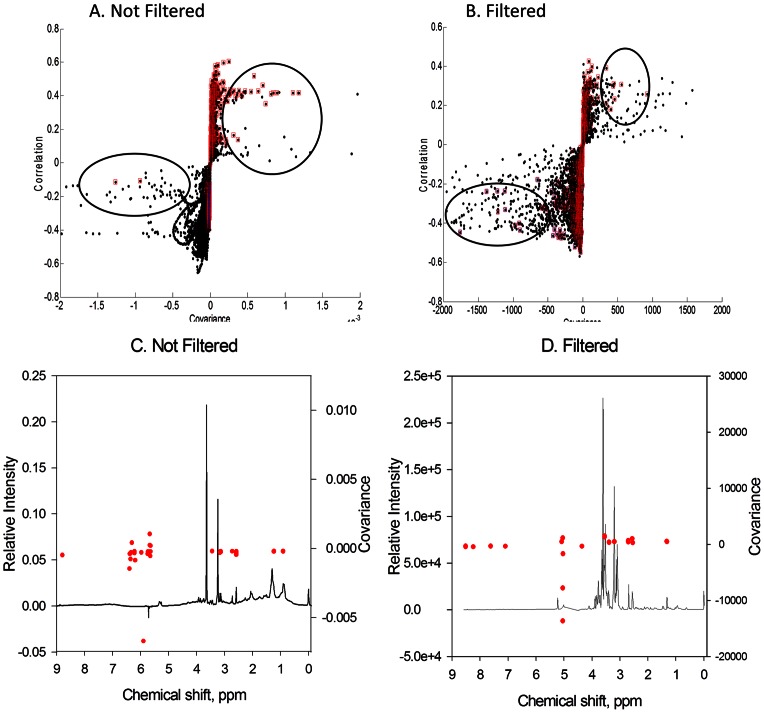
S-plots from multilevel PLS-DA show that ^1^H-NMR signals from many metabolites contribute to separation of vitamin B6 adequate and vitamin B6 restricted conditions. Panels A and B: S-plots that respectively correspond with the score plots in Figure 1A (Not Filtered) and Figure 1B (Filtered) plasma. In these panels, the top 5% of metabolites that contribute to 95% of the separation of baseline and restricted samples are highlighted in red squares. Red-framed points within the ovals represent the most highly discriminating signals. Panels C and D: loading plots illustrating discriminating spectral features from S-plots. Covariance of each discriminating feature is superimposed on the corresponding NMR chemical shift on mean spectra.

Multilevel PLS-DA [54], which is an approach to multivariate analysis that incorporates provisions for paired data, showed distinct separation according to vitamin B-6 status in both unfiltered and filtered plasma (Figure 1). For unfiltered plasma, the first orthogonal component (40% of total variation) and the first principal components explained 40% and 30% of the total variation, respectively. For filtered plasma the first orthogonal component and the first principal component explained 29% and 60% of total variation, respectively. S-plots derived from this analysis of^1^H-NMR data in the multilevel PLS-DA allowed detection of the spectral regions that discriminated between vitamin B-6 adequacy (baseline) and restricted conditions for both filtered and unfiltered plasma (Figure 2A and 2B). When discriminating features were superimposed on mean spectra (Figure 2C and 2D), the discriminating regions appeared to be primarily attributable to lipids, organic acids and amino acids for unfiltered plasma and organic acids, and amino acids for filtered plasma (Figure 2C and 2D).

#### Vitamin B-6 restriction affects the plasma metabolite profile

Analysis using NMR spectral fitting provided an estimation of the concentration of selected plasma constituents for which methods of targeted quantitative analysis were not readily available in this study. We report here this analysis using Chenomx software for acetate, acetoacetate, aspartate, asparagine, choline, formate, fumarate, glutamate, glutamine, lactate, myo-inositol, 2-oxoglutarate, pyruvate, succinate taurine and trimethylamine oxide in a subset (n = 18) of the participants (Table 1). Local hypothesis testing of changes in the estimated plasma concentrations derived from spectral features attributed to acetate, asparagine, glutamine, myo-inositol, lactate, 2-oxoglutarate, pyruvate, taurine and trimethylamine-N-oxide significantly increased (adjusted P<0.05), while that of glutamate significantly decreased (adjusted P<0.05) (Table 1).

**Table 1:** Estimated concentration of selected plasma constituents before and after vitamin B-6 restriction determined in filtered plasma by Chenomx spectral fitting analysis of 1H-NMR.

Variable	Baseline	Vitamin B-6 Restricted	*Adjusted P* 2
	*mmol/L*		
Acetate	56±23	79±49*	0.028
Acetoacetate	49±38	42±28	0.493
Acetone	36±32	39±27	0.317
Asparagine	50±23	75±33*	0.011
Aspartate	49±27	63±30	0.055
Choline	36±33	37±24	0.448
Formate	61±18	79±48	0.158
Glutamate	78±37	61±26*	0.011
Glutamine	130±150	205±201*	<0.001
Myo-Inositol	23±12	39±21*	0.011
2-Oxoglutarate	37±14	51±21*	<0.001
Pyruvate	94±54	121±61*	0.028
Succinate	26±13	30±16	0.305
Trimethylamine-N-oxide	9±10	17±16*	0.004
Urea	1250± 1180	1130±884	0.493

1 Mean ± SD, n = 18. Concentrations approximated from the respective singlet resonances using Chenomx spectral fitting analysis.

2 Adjusted P-value through controlling positive FDR after paired t-tests on changes on log2 transformed concentrations.* designates significant difference at P<0.05. The effect of vitamin B-6 restriction on the overall pattern of constituents in Chenomx analysis was significant in multivariate testing, P = 5.3×10^−7^.

The evaluation of the ratios of several of these plasma constituents allowed us to probe possible biochemical effects of vitamin B-6 restriction. Whereas spectral features attributable to asparagine and glutamine concentrations increased (Table 1), the ratios reflecting the relative extent of amidation differed (Table 2). Vitamin B-6 restriction did not significantly change the asparagine/aspartate ratio, but the ratio of glutamine/glutamate more than doubled due to the restriction (P<0.001). In addition, the ratio of the 2-ketoacids pyruvate and 2-oxoglutarate and the corresponding amino acids alanine and glutamate reflected the balance of respective aminotransferase reactions. The pyruvate/alanine ratio did not change significantly, while the 2-ketoglutarate/glutamate ratio increased 76% due to vitamin B-6 restriction (P<0.001). The ratio of plasma lactate/pyruvate, which is indicative of cytoplasmic redox state and cellular respiration, did not change due to vitamin B-6 restriction (8.3±2.0 versus 7.6±1.5; P = 0.242).

**Table 2:** Ratios of selected plasma constituents determined in filtered plasma by 1H-NMR, before and after vitamin B-6 restriction.

Variables	Baseline	Vitamin B-6 Restricted	*P*
Transamination ratios			
Pyruvate/Alanine	0.280±0.093	0.296±0.095	0.554
2-Oxoglutarate/Glutamate	0.556±0.303	0.984±0.582*	0.001
Amidation ratios			
Asparagine/Aspartate	1.20±0.56	1.39±0.66	0.250
Glutamine/Glutamate	1.42±1.23	3.17±2.76*	0.001

1 Mean ± SD, n = 18.

2 *P* value determined by paired t-test after log_2_ transformation. * designates a significant difference at P<0.05.

The concentration of amino acids and glutathione determined by targeted quantitative methods (tandem mass spectrometry, GC-MS and HPLC) revealed significant effects of vitamin B-6 restriction (Table 3). Plasma cystathionine, which is a functional biomarker of vitamin B-6 deficiency indicating functional impairment of the transsulfuration pathway [27], [37], [38], increased markedly (adjusted P = 0.0002). Plasma proline exhibited an unexpected increase (adjusted P<0.05), but there was no significant change in most of the other amino acids evaluated (alanine, arginine, asx, citrulline, glx, glycine, histidine, leucine + isoleucine, methionine, ornithine, phenylalanine, serine, tyrosine, and valine). In this context, asx and glx refer to the concentrations of aspartate and glutamate plus a contribution from partial hydrolysis of their amides, asparagine and glutamine, under the conditions of the tandem mass spectral analysis. The concentration of plasma glucose measured enzymatically was not changed by vitamin B-6 restriction (4.71±0.676 versus 4.52±0.543 mmol/L; adjusted P = 0.122). We found no change in the concentrations of 45 acylcarnitines or in total acylcarnitines (Table S2).

**Table 3:** Concentration of plasma free amino acids and glutathione determined by targeted quantitative methods before and after vitamin B-6 restriction.^1^

Variable	Method	Baseline	Vitamin B-6 Restricted	*Adjusted P* 2
		*µmol/L*	*µmol/L*	
Alanine	Tandem MS	377±112	396±77	0.281
Arginine	Tandem MS	94±26	86±17	0.281
Asx	Tandem MS	77±21	74±16	0.577
Citrulline	Tandem MS	33±10	34±9	0.577
Cystathionine	GC-MS	131±65	199±56*	<0.001
Cysteine (total)	HPLC	256±39	253±36	0.577
Glx	Tandem MS	73±24	82±32	0.281
Glutathione (total)	HPLC	7.17±1.84	7.15±1.26	0.855
Glycine	Tandem MS	357±83	393±95	0.281
Histidine	Tandem MS	82±13	77±18	0.281
Homocysteine (total)	HPLC	6.97±1.26	6.97±1.33	0.925
Leucine/Isoleucine	Tandem MS	158±31	154±27	0.577
Methionine	Tandem MS	25±6	26±6	0.724
Ornithine	Tandem MS	47±11	49±12	0.483
Phenylalanine	Tandem MS	78±21	74±19	0.281
Proline	Tandem MS	190±90	223±84*	0.004
Serine	Tandem MS	115±24	125±33	0.281
Tyrosine	Tandem MS	75±17	71±13	0.329
Valine	Tandem MS	196±34	190±29	0.565

1 Mean ± SD, n = 23.

2 Adjusted P-value through controlling positive FDR after paired t-tests on changes on log2 transformed concentrations. * designates significant difference at P<0.05. The effect of vitamin B-6 restriction on the overall pattern of constituents was significant in multivariate testing, *P* = 0.0296.

We also evaluated whether the change in the various quantified plasma constituents was related to the change in plasma PLP concentration. No significant relationship with PLP was found for the amino acids and glutathione (adjusted P = 0.764) or for the acylcarnitines (adjusted P = 0.747).

## Discussion

### Effectiveness of the vitamin B-6 restriction protocol

The controlled vitamin B-6 restriction protocol employed in this study provided a novel and reproducible tool for investigating metabolic effects of marginally deficient vitamin B-6 status [24]–[28]. We recognize, however, that the short term metabolic effects of marginal vitamin B-6 status may not reflect the mechanisms responsible for the association of chronic low vitamin B-6 status with greater long-term risk of various forms of chronic disease. We also recognize that subtle differences between the controlled diet and the previous self-selected diet of the participants could contribute to observed metabolite patterns. This protocol constituted an opportunity in which to investigate the utility of metabolomic approaches in the context of controlled low vitamin B-6 status over a relatively brief period. To our knowledge, this is the first report of the use of combined global and targeted metabolomic approaches for probing micronutrient deficiency of any type.

### Metabolomic tools in vitamin B-6 research

The combined NMR and targeted mass spectral analysis in this study yielded new insights into the metabolic effects of vitamin B-6 restriction. They also indicate the merits of concurrently using NMR and mass spectrometry in nutritional metabolomics. ^1^H-NMR has several analytical strengths when investigating micronutrient status. First, NMR can detect differences in overall plasma composition in both whole (unfiltered) plasma and following filtration to remove most proteins, lipids and lipoproteins. Minimal sample preparation is required, and aspects of the procedure are suitable for automation. Second, because of the comparatively low sensitivity of ^1^H-NMR (relative to mass spectrometry-based procedures), the spectra reflect the metabolic phenotype at the level of macronutrients and their metabolites. This provides a particularly useful tool in evaluating the widespread effects of its micronutrient restriction on human metabolism. The focus of ^1^H-NMR on relatively high-concentration plasma constituents, such as amino acids and organic acids, yields a broad assessment of the direct and indirect effects of vitamin B-6 restriction on human intermediary metabolism. Third, our results show that ^1^H-NMR has potential as a diagnostic technique that compliments conventional analysis of a single biomarker (e.g., plasma PLP) in characterizing marginal vitamin B-6 deficiency. Further investigation will be needed to validate this diagnostic application more fully.

Based on the current data, the sensitivity and specificity of multilevel PLS-DA of NMR spectra in reflecting overall metabolic effects of vitamin B-6 restriction are excellent. The minimal sample preparation needed for NMR analysis preserves the patterns of labile compounds such as asparagine and glutamine. However, the estimation of metabolite concentrations by spectral fitting using techniques such as illustrated here with Chenomx software should not be viewed as quantitatively conclusive. Confirmatory targeted analysis should be conducted wherever possible, especially for these metabolites in which changes are subtle. In the case of free amino acids such as asparagine, aspartate, glutamate and glutamine, differences between NMR-based and targeted mass spectrometry-based analyses may partially be attributable to NMR response to amino acid residues in the plasma peptides [55] that would be present in the ultrafiltrates used in NMR-Chenomx analysis. Other sources of variability in metabolite quantification by spectral fitting have been reported [56].

### Metabolic consequences of vitamin B-6 restriction

Because pyridoxal phosphate serves a coenzymatic function in over 140 reactions, the potential for metabolic effects of vitamin B-6 deficiency extends broadly through many phases of human metabolism. As illustrated by our previous studies involving the transsulfuration enzymes cystathionine β-synthase and cystathionine γ-lyase [26], [28], PLP binding affinity is not fully informative of impairment caused by vitamin B-6 restriction. Broadly focused metabolite profiling methods allow one to assess more fully the extended effects of a nutritional condition, such as marginal vitamin B-6 deficiency.

Many reactions of the interconversion and catabolism of amino acids require PLP. PLP-dependent aspects of one-carbon metabolism (cytoplasmic and mitochondrial serine hydroxymethyltransferases and the mitochondrial glycine cleavage system) that regulate these amino acids play an important role in the supply and balance of 1C units in metabolism [25], [27]. Applications of in vivo stable isotopic tracer kinetics and mathematical modeling have shown situations in which in vivo fluxes of PLP-dependent processes and substrate/product concentrations are not totally predicted by vitamin B-6 status. For example, although the in vitro activities of cytoplasmic serine hydroxymethyltransferase (SHMT) and mitochondrial SHMT are strongly influenced by vitamin B-6 status [25], [57], in vivo SHMT flux is relatively resilient to marginal deficiency [25], [58]. Likewise, overall transsulfuration flux and cysteine production are maintained during marginal vitamin B-6 deficiency [26], [28] despite the sensitivity of the cystathionine γ-lyase reaction to cellular PLP deficiency and resulting buildup of cystathionine in tissues and plasma [27], [37], [38]. The PLP-dependent mitochondrial glycine cleavage system accounts for approximately 20 times more 5,10-methylenetetrahydrofolate production than needed for cellular methyl synthesis demands [59]. The increased glycine concentration frequently observed in marginal vitamin B-6 deficiency presumably reflects substrate accumulation due to reduced activity of the glycine cleavage system [25], [27], [58]. However, in vivo tracer kinetic results indicate no significant reduction in glycine cleavage system flux from vitamin B-6 restriction [27].

The spectral features reflecting substantial increases in asparagine and glutamine and the marked increase in the glutamine/glutamate ratio (Table 2) observed here were unexpected. Neither glutamine synthetase, which catalyzes ATP-dependent condensation of glutamate and ammonia, nor asparagine synthetase, which catalyzes the ATP-dependent transfer of the terminal amide group of glutamine to aspartate, requires PLP, nor do the catabolic enzymes glutaminase and asparaginase. In view of the importance of inter-organ transport of glutamine and the regulatory role of glutaminases [60], [61], our findings suggest fundamental perturbations in the inter-organ trafficking of these amino acids may be responsible for the changes in their plasma concentrations associated with vitamin B-6 restriction. The unexpected increase in plasma proline suggests that it may be a secondary consequence of the altered glutamate and glutamine concentrations because proline synthesis occurs by way of pyrroline 5-carboxylate [60]. It is likely that the changes in plasma myo-inositol and trimethylamine oxide were mainly attributable to differences in diet composition from the participants' pre-study diets, because their metabolism does not directly involve PLP-dependent processes.

The activity and extent of coenzyme saturation of PLP-dependent aminotransferases has long been recognized and used diagnostically in assessing vitamin B-6 status [62]. However, the impact of vitamin B-6 insufficiency on the profile of tissue and plasma amino acids and, particularly, the sensitivity of such reactions in maintaining anaplerotic reactions remains unclear. Increases in plasma concentrations of pyruvate and 2-oxoglutarate may reflect reduced utilization of 2-ketoacids in aminotransferase reactions or in other phases of metabolism. The lack of a significant change in formate concentration is consistent with findings from an NMR study in rats, in which deficiency of vitamin B-12, but not B-6 deficiency, caused an increase in plasma and urinary formate [63]. Changes in the profile of tryptophan catabolic products in the kynurenine pathway are associated with vitamin B-6 deficiency [10], [64], [65]. Targeted analysis has revealed such changes in tryptophan metabolites in the present study (reported separately).

We have previously shown that vitamin B-6 deficiency is associated with markedly increased total glutathione concentration in rat liver in direct proportion to the severity of the deficiency [66] and in altered glutathione synthesis in a subset of vitamin B-6 restricted healthy humans [67]. These responses may be due to oxidative stress associated with vitamin B-6 deficiency [68]–[70] independent of substrate availability, a hypothesis that has been supported by mathematical modeling [58]. The role of such oxidative stress and its impact on the plasma metabolome during vitamin B-6 deficiency is presently unknown.

## Conclusions

This study demonstrates that our vitamin B-6 restriction protocol induces fundamental changes in the plasma metabolite profile, strongly suggesting that marginal vitamin B-6 deficiency exerts far-reaching effects on human metabolism. These observations extend our understanding of the impact and interpretation of the marginal nutritional status and will aid in hypothesis development in the design of future research regarding the mechanistic relationship between low vitamin B-6 status and chronic disease.

## Supporting Information

Figure S1 **Representative ^1^H-NMR spectra of unfiltered and filtered plasma from a single participant shown before and after vitamin B-6 restriction, with signals from EDTA designated.** Small arrows designate visually apparent differences in certain spectral features of these spectra.
(TIF)

Table S1 **Baseline characteristics of 23 healthy men and women participating in the study.**
(DOCX)

Table S2 **Concentration of individual and total acylcarnitines in plasma determined by tandem mass spectrometry before and after vitamin B-6 restriction.**
(DOCX)
