# Supplementary figures and images for: Metabolomic Analysis Reveals Extended Metabolic Consequences of Marginal Vitamin B-6 Deficiency in Healthy Human Subjects
Source: PLoS One. 2013 Jun 11;8(6):e63544. doi: 10.1371/journal.pone.0063544 (PMC3679127; doi:10.1371/journal.pone.0063544)

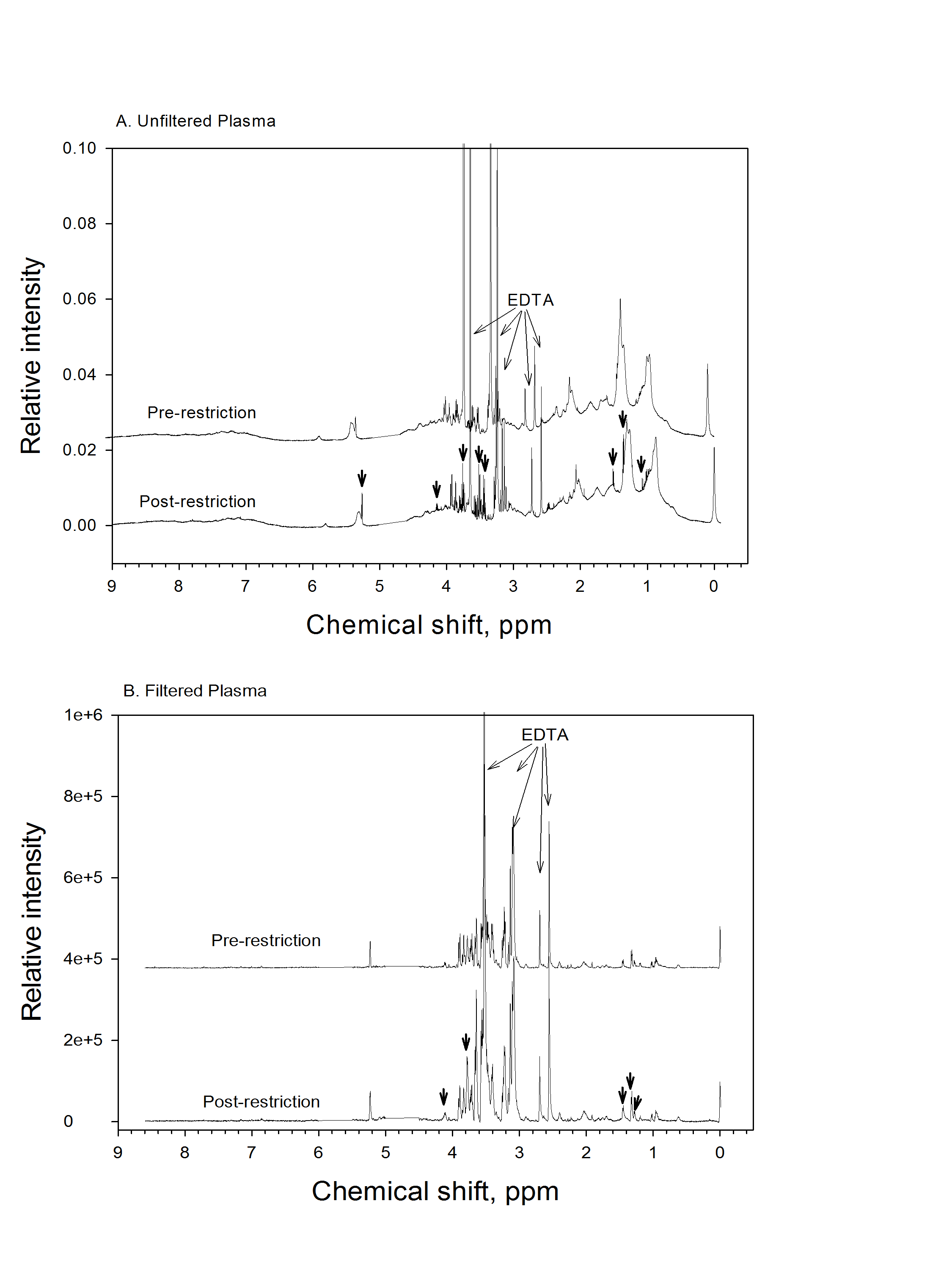

Supplement: Figure S1 — Representative 1H-NMR spectra of unfiltered and filtered plasma from a single participant shown before and after vitamin B-6 restriction, with signals from EDTA designated. Small arrows designate visually apparent differences in certain spectral features of these spectra. (TIF) [file pone.0063544.s001.tif]
