# Supplementary material for: Metabolomic Analysis Reveals Extended Metabolic Consequences of Marginal Vitamin B-6 Deficiency in Healthy Human Subjects
Source: PLoS One. 2013 Jun 11;8(6):e63544. doi: 10.1371/journal.pone.0063544 (PMC3679127; doi:10.1371/journal.pone.0063544)
Supplement: Table S1 — Baseline characteristics of 23 healthy men and women participating in the study. (DOCX) [file pone.0063544.s002.docx]

**Supporting Material**

Supporting Table S1. Baseline characteristics of 23 healthy men and women participating in the study.

| **Characteristic** | **Men** | **Women** | **Total** |
| --- | --- | --- | --- |
|  | (n = 12) | (n = 11) | (n = 23) |
| Racial/Ethnic Distribution (n) |  |  |  |
| African American | 2 | 1 | 3 |
| Asian | 1 | 1 | 2 |
| Caucasian | 6 | 6 | 12 |
| Hispanic | 3 | 3 | 6 |
| Age (y) | 24 ± 5*^1^* | 25 ± 6 | 25 ± 5 |
| BMI (kg/m^2^) | 24.5 ± 2.7 | 23.6 ± 2.3 | 24.1 ± 2.5 |
| Plasma PLP (nmol/L) | 59 ± 13 | 45 ± 13^2^ | 52 ± 14 |
| Serum folate (nmol/L) | 34 ± 8 | 30 ± 7 | 32 ± 7 |
| Serum vitamin B-12 (pmol/L) | 349 ± 115 | 388 ± 129 | 368 ± 124 |
| Plasma homocysteine (µmol/L) | 7.5 ± 1.0 | 6.4 ± 1.3 | 7.0 ± 1.3 |

*^1^*All values are means ± SDs. These data have been reported previously [27,28,29].

*^2^*Significant difference between men and women, *P* < 0.05 (2-sample t-test).
