# Supplementary material for: Metabolomic Analysis Reveals Extended Metabolic Consequences of Marginal Vitamin B-6 Deficiency in Healthy Human Subjects
Source: PLoS One. 2013 Jun 11;8(6):e63544. doi: 10.1371/journal.pone.0063544 (PMC3679127; doi:10.1371/journal.pone.0063544)
Supplement: Table S2 — Concentration of individual and total acylcarnitines in plasma determined by tandem mass spectrometry before and after vitamin B-6 restriction. (DOCX) [file pone.0063544.s003.docx]

**Supporting Material**

Supporting Table S2. Concentration of individual and total acylcarnitines in plasma determined by tandem mass spectrometry before and after vitamin B-6 restriction.

| **Variable** | **Baseline** | **Restricted** | | ***Adjusted P*** ^2^ | |  |
| --- | --- | --- | --- | --- | --- | --- |
|  | * μmol/L* | |  | | |  |
| C2 | 5.92±2.65 | 5.63±1.83 | | 0.940 | | |
| C3 | 0.383±0.203 | 0.384±0.189 | | 0.940 | | |
| C4/Ci4 | 0.127±0.078 | 0.108±0.056 | | 0.215 | | |
| C5:1 | 0.118±0.091 | 0.118±0.100 | | 0.907 | | |
| C5's | 0.110±0.082 | 0.094±0.039 | | 0.773 | | |
| C4-OH | 0.025±0.013 | 0.028±0.010 | | 0.125 | | |
| C6 | 0.015±0.022 | 0.015±0.030 | | 0.415 | | |
| C5-OH/C3-DC | 0.053±0.078 | 0.084±0.087 | | 0.113 | | |
| Ci4-DC/C4-DC | 0.045±0.024 | 0.043±0.012 | | 0.221 | | |
| C8:1 | 0.182±0.073 | 0.236±0.105* | | 0.076 | | |
| C8 | 0.069±0.037 | 0.075±0.036 | | 0.685 | | |
| C5-DC | 0.042±0.029 | 0.033±0.019 | | 0.238 | | |
| C6:1-DC/C8:1-OH | 0.021±0.013 | 0.022±0.012 | | 0.940 | | |
| C6-DC | 0.051±0.028 | 0.062±0.018 | | 0.105 | | |
| C10:3 | 0.059±0.038 | 0.083±0.037 | | 0.105 | | |
| C10:2 | 0.021±0.024 | 0.024±0.030 | | 0.940 | | |
| C10:1 | 0.123±0.051 | 0.124±0.050 | | 0.907 | | |
| C10 | 0.171±0.090 | 0.169±0.099 | | 0.940 | | |
| C7-DC | 0.013±0.025 | 0.004±0.009 | | 0.451 | | |
| C8:1-DC | 0.019±0.012 | 0.019±0.008 | | 0.247 | | |
| C10-OH/C8-DC | 0.026±0.022 | 0.029±0.015 | | 0.207 | | |
| C12:1 | 0.059±0.033 | 0.063±0.024 | | 0.415 | | |
| C12 | 0.050±0.033 | 0.048±0.025 | | 0.940 | | |
| C12-OH/C10-DC | 0.004±0.003 | 0.002±0.002 | | 0.415 | | |
| C14:2 | 0.031±0.028 | 0.031±0.016 | | 0.390 | | |
| C14:1 | 0.054±0.029 | 0.056±0.024 | | 0.608 | | |
| C14 | 0.019±0.014 | 0.020±0.012 | | 0.521 | | |
| C14:1-OH/C12:1-DC | 0.010±0.006 | 0.009±0.004 | | 0.940 | | |
| C14-OH/C12-DC | 0.007±0.003 | 0.006±0.003 | | 0.824 | | |
| C16:2 | 0.005±0.003 | 0.004±0.002 | | 0.940 | | |
| C16:1 | 0.012±0.006 | 0.012±0.004 | | 0.415 | | |
| C16 | 0.055±0.016 | 0.055±0.012 | | 0.940 | | |
| C16:1-OH/C14:1-DC | 0.006±0.002 | 0.005±0.002 | | 0.940 | | |
| C16-OH/C14-DC | 0.003±0.002 | 0.003±0.002 | | 0.940 | | |
| C18:2 | 0.041±0.017 | 0.035±0.009 | | 0.275 | | |
| C18:1 | 0.076±0.026 | 0.086±0.021 | | 0.207 | | |
| C18 | 0.035±0.012 | 0.035±0.011 | | 0.758 | | |
| C18:2-OH | 0.003±0.004 | 0.003±0.004 | | 0.824 | | |
| C18:1-OH/C16:1-DC | 0.004±0.003 | 0.005±0.003 | | 0.940 | | |
| C18-OH/C16-DC | 0.004±0.004 | 0.004±0.002 | | 0.907 | | |
| C20:4 | 0.005±0.003 | 0.005±0.003 | | 0.907 | | |
| C20 | 0.004±0.002 | 0.003±0.002 | | 0.907 | | |
| C20:1-OH/C18:1-DC | 0.004±0.003 | 0.007±0.004 | | 0.238 | | |
| C20-OH/C18-DC | 0.007±0.003 | 0.009±0.007 | | 0.238 | | |
| C22 | 0.003±0.003 | 0.004±0.002 | | 0.415 | | |
| Total acylcarnitines | 8.09±2.94 | 7.89±2.12 | | 0.916 | | |
|  |  |  | |  |  |  |

^1^ Mean ± SD, n=23. Abbreviations: C = carbon number of acyl chain; number after colon designates double bonds; DC = dicarboxy; OH = hydroxy.

^2^ *A*djusted P-value through controlling FDR by the positive FDR method after paired t-tests on changes on log_2_ transformed concentrations. There was no significant effect of vitamin B-6 restriction in multivariate testing (P=0.106) and no significant difference in total acylcarnitines (P=0.916).
